# Supplementary material for: MCM double hexamer loading visualized with human proteins
Source: Nature. 2024 Nov 27;636(8042):499–508. doi: 10.1038/s41586-024-08263-6 (PMC11634765; doi:10.1038/s41586-024-08263-6)
Supplement: Supplementary file 1 — Supplementary Figs. 1–4. [file 41586_2024_8263_MOESM1_ESM.pdf]

nature portfolio

https://doi.org/10.1038/s41586-024-08263-6

Supplementary information

MCM double hexamer loading visualized with human proteins

In the format provided by the authors and unedited

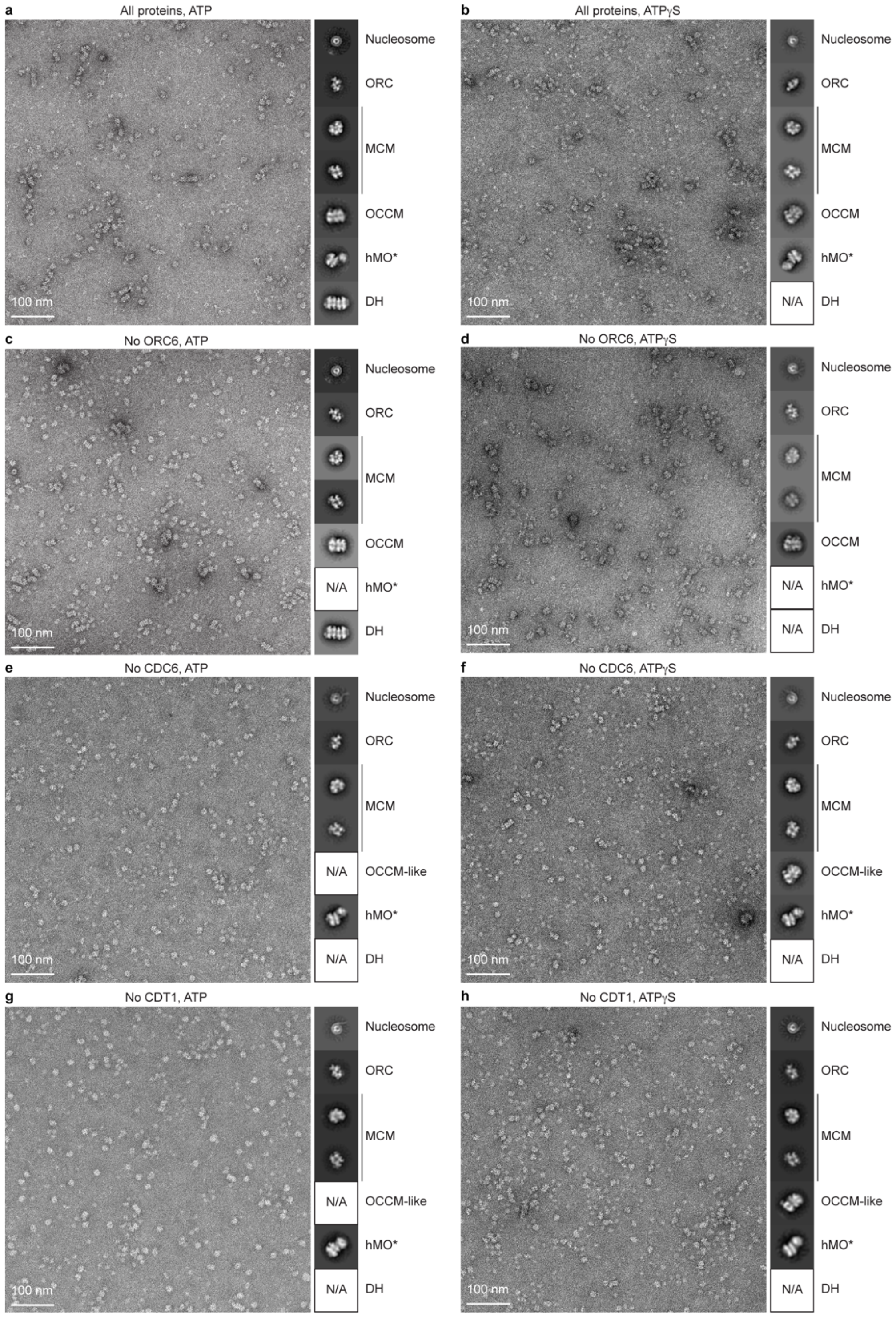

Supplementary Fig. 1 Representative negative-stain EM micrographs and 2D averages

of human MCM loading reactions (△N) performed in EM buffer showing particles observed under the indicated reaction conditions.

a, Nucleosomes, ORC, MCM, OCCM, hMO* and DH classes were observed in the presence of ATP and all proteins. **b**, All complexes apart from DH were present when the slowly hydrolysable ATP analogue, ATPγS, was used. **c, d**, Without ORC6, DH classes were visible in the presence of ATP (**c**). No hMO* had formed when ORC6 was omitted, either in the presence of ATP (**c**) or ATPγS (**d**). **e,** No DH were observed in the absence of CDC6 in ATP under these conditions. **f**, In ATPγS conditions, OCCM-like and hMO* class averages were visible in the absence of CDC6. **g**, DH formation depends on CDT1 in ATP conditions. **h**, Similar to the experiments without CDC6, OCCM-like and hMO* class averages were detected when CDT1 was omitted in ATPγS conditions.

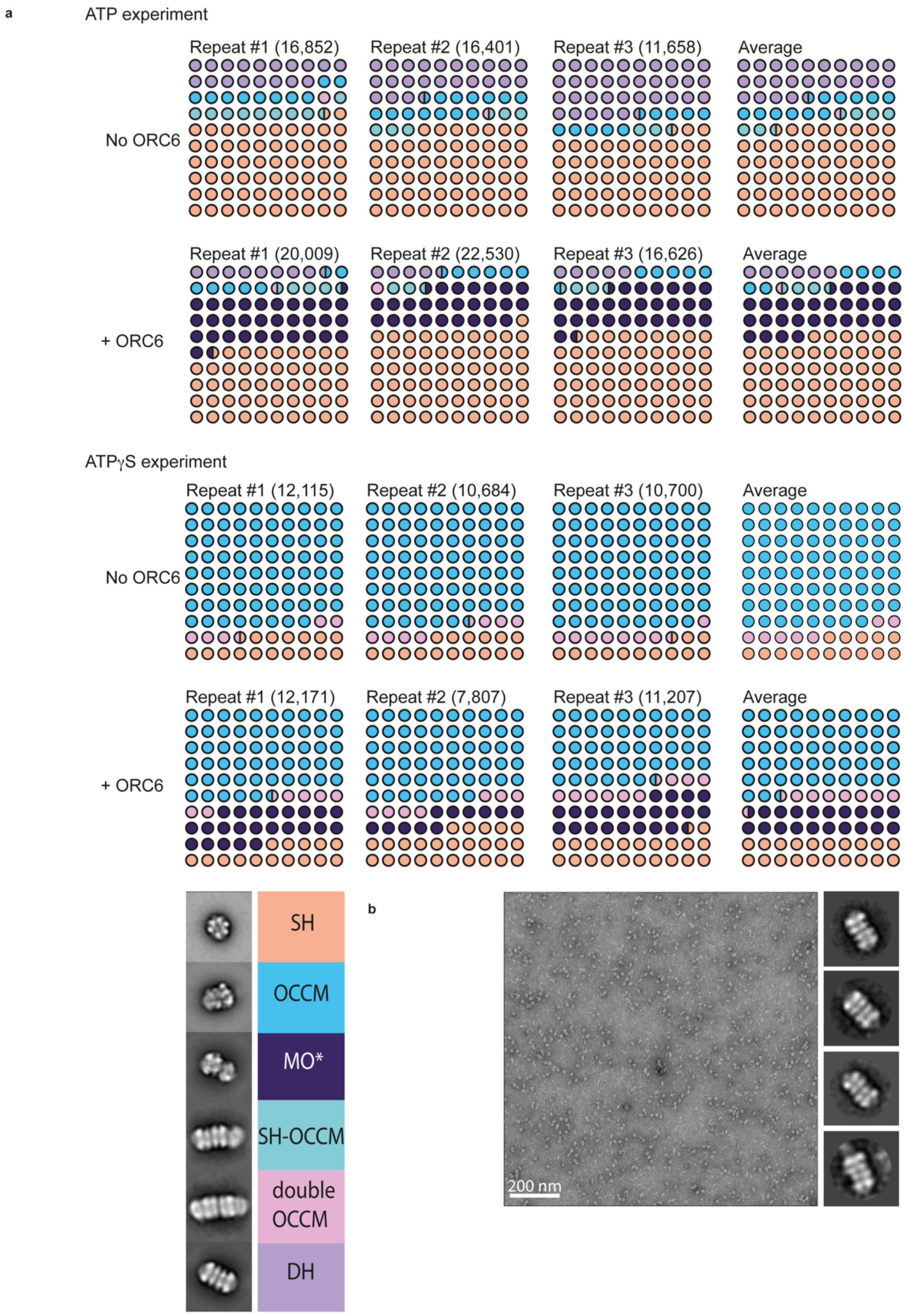

Supplementary Fig. 2 Negative stain EM analysis of hDH loading without ORC6 and Cdc6.

(a) ORC6 dropout experiment in ATP and ATPγS. Three individual MCM loading reactions were carried out per experimental condition (+/- ORC6 in ATP or ATPgS) and 2D classes of all MCM species were calculated. The amount of different MCM species (DH = violet, single OCCM = cyan, double OCCM = pink, SH-OCCM = turquois, MO* = dark blue, single MCM = peach) are represented as fraction of all well-averaging particles in a 10x10 dot plot (1 dot corresponding to 1% of well-averaging particles) for each repeat and as average per condition. Representative 2D classes are highlighted for each detected MCM species. **(b)** CDC6 dropout experiment. Representative negative-stain micrograph of a MCM double hexamer loading reaction in absence of CDC6 and ORC6 (scale bar = 200 nm). 2D class averages of the human MCM double hexamer were detected under these conditions.

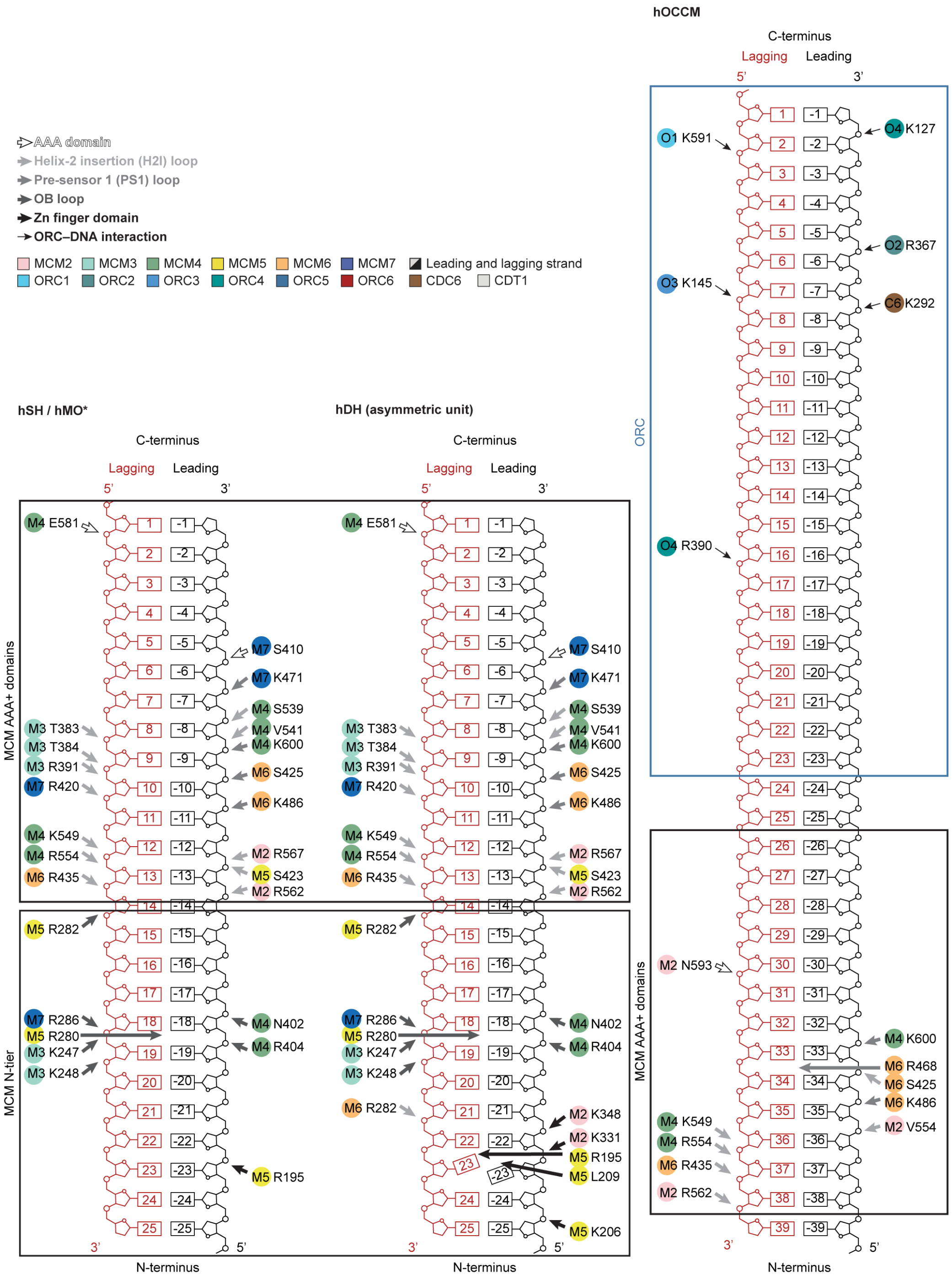

Supplementary Fig.3 Protein–DNA interactions within the hSH, hMO*, hDH (asymmetric unit) and hOCCM.

Source data

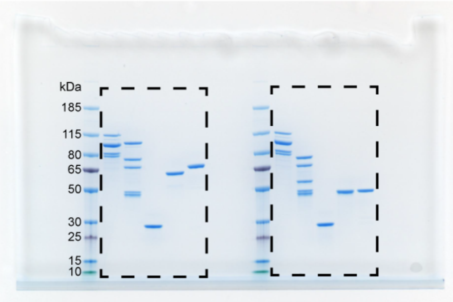

Fig. 1a (left) Fig. 1a (right)

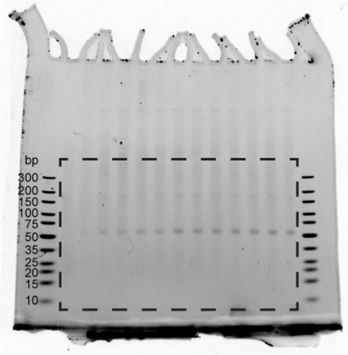

Fig. 1c (top)

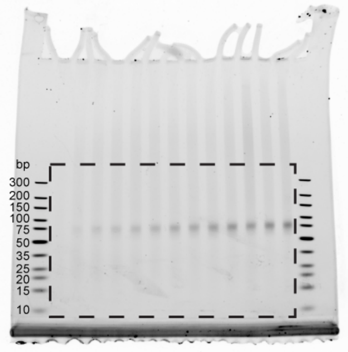

Fig. 1c (bottom)

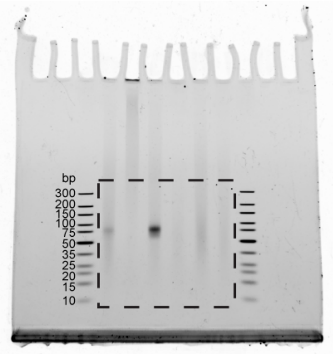

Fig. 1d

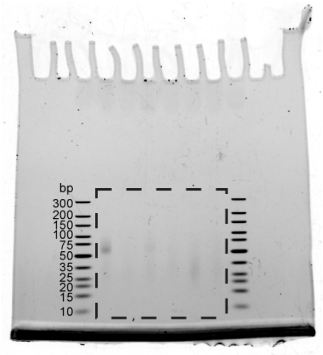

Fig. 1e

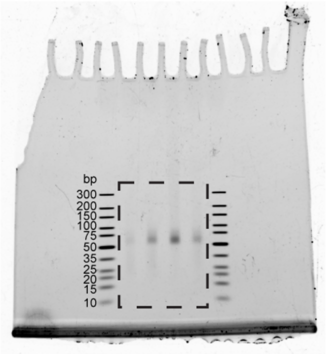

Fig. 1f

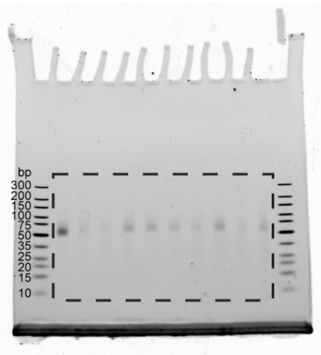

Fig. 1g

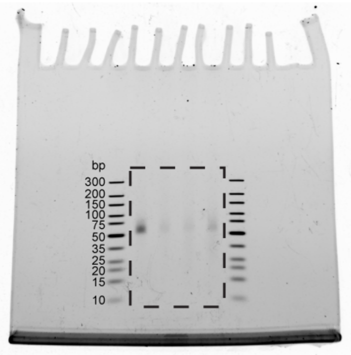

Fig. 1h

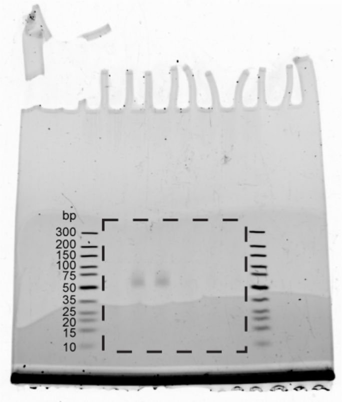

Fig. 1i

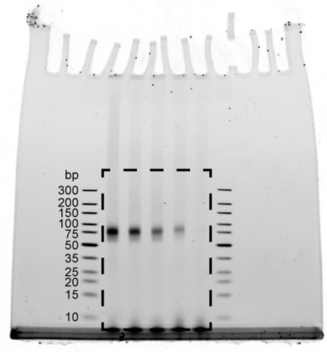

Fig. 1j

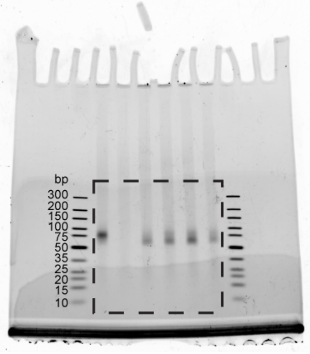

Fig. 2e

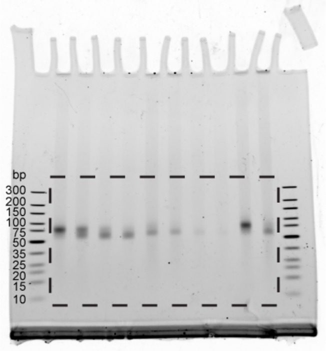

Fig. 2g

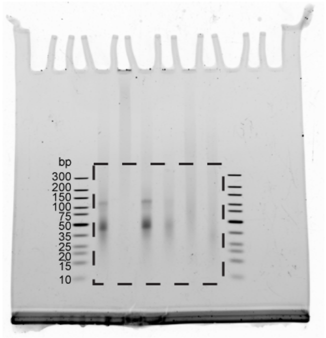

Fig. 3a

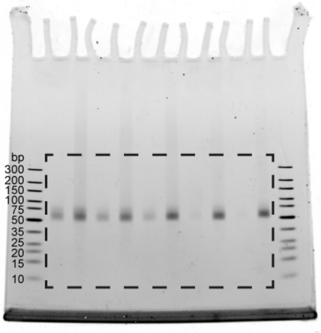

Fig. 3f

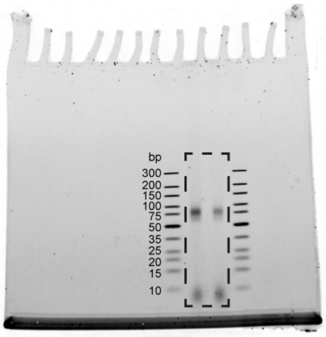

Fig. 3g

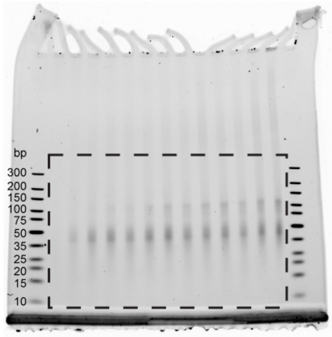

Fig. 3i

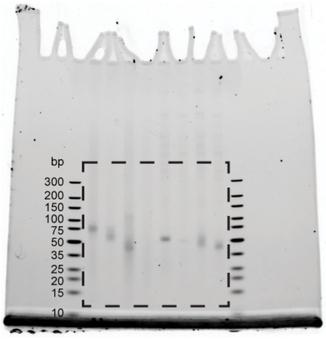

Fig. 3k

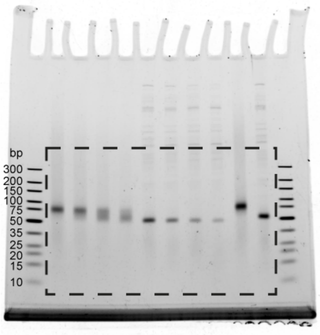

ED Fig. 1b

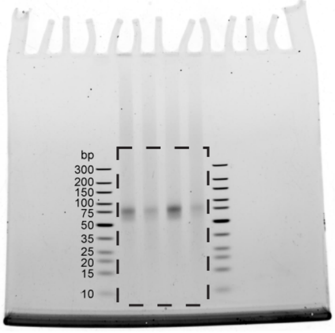

ED Fig. 1c

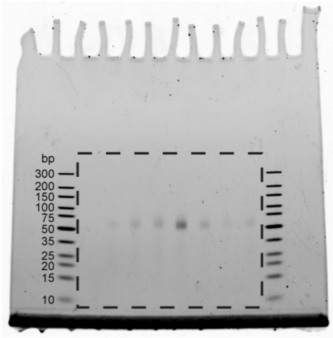

ED Fig. 1d

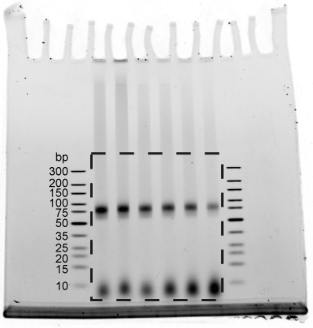

ED Fig. 1e

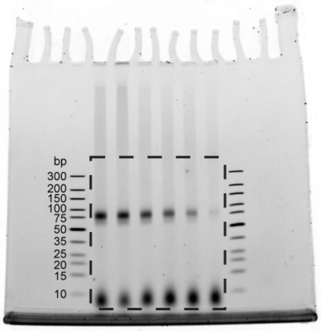

ED Fig. 1f

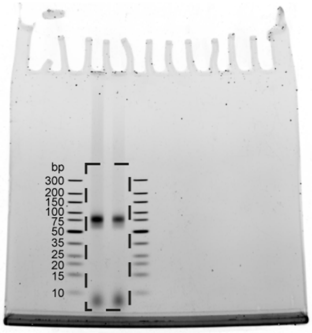

ED Fig. 1g (gel 1)

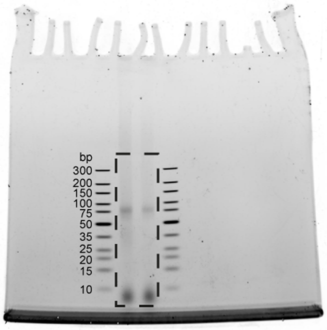

ED Fig. 1g (gel 2)

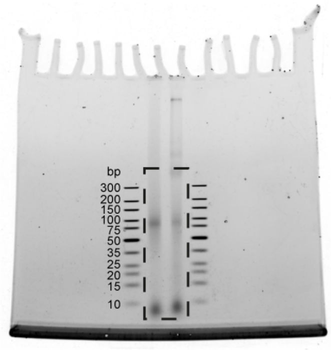

ED Fig. 1g (gel 3)

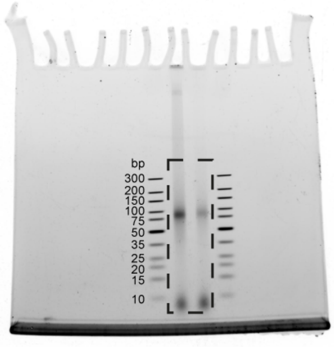

ED Fig. 1g (gel 4)

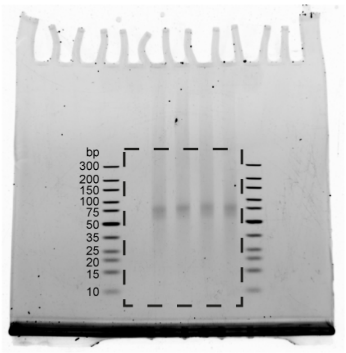

ED Fig. 1h

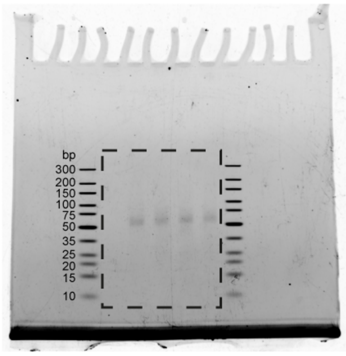

ED Fig. 1i

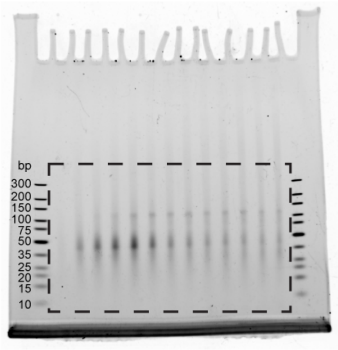

ED Fig. 1m (top)

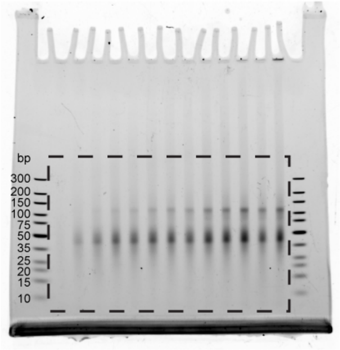

ED Fig. 1m (bottom)

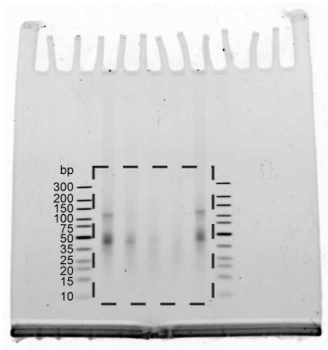

ED Fig. 1n

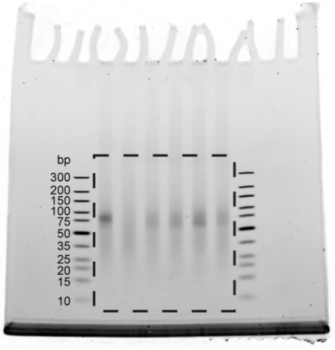

ED Fig. 3d

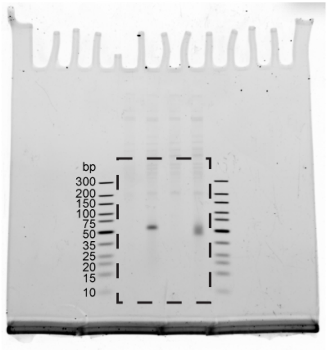

ED Fig. 4a

Supplementary Fig. 4 Source data.
